# Genome-wide Phenotypic Profiling Identifies and Categorizes Genes Required for Mycobacterial Low Iron Fitness

**DOI:** 10.1038/s41598-019-47905-y

**Published:** 2019-08-06

**Authors:** Marte S. Dragset, Thomas R. Ioerger, Yanjia J. Zhang, Mali Mærk, Zekarias Ginbot, James C. Sacchettini, Trude H. Flo, Eric J. Rubin, Magnus Steigedal

**Affiliations:** 1 NTNU Norwegian University of Science and Technology, Centre of Molecular Inflammation Research and Department of Clinical and Molecular Medicine, Trondheim, 7491 Norway; 2 Harvard T.H. Chan School of Public Health, Department of Immunology and Infectious Diseases, Boston, MA 02115 USA; 3 Germans Trias i Pujol Research Institute, Tuberculosis Research Unit, Badalona, 80916 Spain; 4 Texas A&M University, Department of Computer Science, College Station, TX 77843 USA; 5 Texas A&M University, Department of Biochemistry and Biophysics, College Station, TX 77843 USA; 6 St. Olavs University Hospital, Department of Medical Microbiology, Trondheim, 7030 Norway

**Keywords:** Bacterial genes, Pathogens

## Abstract

Iron is vital for nearly all living organisms, but during infection, not readily available to pathogens. Infectious bacteria therefore depend on specialized mechanisms to survive when iron is limited. These mechanisms make attractive targets for new drugs. Here, by genome-wide phenotypic profiling, we identify and categorize mycobacterial genes required for low iron fitness. *Mycobacterium tuberculosis* (*Mtb*), the causative agent of tuberculosis (TB), can scavenge host-sequestered iron by high-affinity iron chelators called siderophores. We take advantage of siderophore redundancy within the non-pathogenic mycobacterial model organism *M*. *smegmatis* (*Msmeg*), to identify genes required for siderophore dependent and independent fitness when iron is low. In addition to genes with a potential function in recognition, transport or utilization of mycobacterial siderophores, we identify novel putative low iron survival strategies that are separate from siderophore systems. We also identify the *Msmeg in vitro* essential gene set, and find that 96% of all growth-required *Msmeg* genes have a mutual ortholog in *Mtb*. Of these again, nearly 90% are defined as required for growth in *Mtb* as well. Finally, we show that a novel, putative ferric iron ABC transporter contributes to low iron fitness in *Msmeg*, in a siderophore independent manner.

## Introduction

Tuberculosis (TB), caused by *Mycobacterium tuberculosis* (*Mtb*), killed 1.6 million humans in 2018^1^. The emerging threat of drug resistant *Mtb* adds burden to an already existing world health problem, and calls for urgent identification of new mycobacterial drug targets. *Mtb* depends on specialized mechanisms to access iron during infection^2–5^. These mechanisms make attractive targets for TB drug development^6,7^. Mycobacteria produce and secrete two types of high affinity iron chelators, siderophores, to scavenge ferric (Fe^3+^) iron; the mycobactins (here referring to both the insoluble membrane-embedded mycobactin and the soluble carboxymycobactin) and exochelin. Exochelin is produced only by rapidly growing, saprophytic mycobacteria like *M*. *smegmatis* (*Msmeg*) and *M*. *neoaurum*, while mycobactins are found in most mycobacterial species. The biosynthesis of siderophores and their transport across the inner membrane is well understood; however, transport across the outer membrane remains largely unknown. In addition to siderophores, mycobacteria can acquire iron by heme uptake (hemophores), sequestration of holo-transferrin and holo-lactoferrin, and through low-affinity porins (mycobacterial iron acquisition is thoroughly reviewed elsewhere^7–10^). *Mtb* is able to persist at low iron levels over a prolonged period of time, and launches a transcriptional response to adapt to iron availability^11^. The iron dependent repressor IdeR regulates one-third of the mycobacterial genes found to be repressed by iron, including genes for siderophore pathways^12^. Another protein, HupB, is shown to positively regulate *Mtb* siderophore biosynthesis in response to iron^13^. Furthermore, low environmental iron affects mycobacterial cell wall integrity (as well as membrane microvesicle production^14^), and the activity of diverse iron-containing enzymes^7,15^. Mechanisms not directly implicated in uptake of iron per se may, thus, play a pivotal role in mycobacterial fitness under iron-limited conditions.

*Msmeg* is widely used to study basic mycobacterial mechanisms, due to its non-pathogenic and fast-growing nature^16^. Pathways that promote low iron proliferation, separate from exochelin, are likely to be conserved within mycobacteria. In fact, we demonstrated the need of ESX-3, a type VII secretion system, for mycobactin-mediated iron uptake using *Msmeg*^17,18^, a finding later confirmed in *Mtb*^19,20^. Here, we take advantage of siderophore redundancy within *Msmeg* and define and categorize genes required for mycobactin, exochelin or siderophore independent modes of low iron growth by transposon insertion sequencing (Tnseq)^21^. (In fact, the power of *Msmeg* siderophore redundancy to identify genetic interaction partners to siderophore pathways was previously demonstrated by Judd *el al*, in a proof-of-principle synthetic genetic array^22^). To accomplish this, we developed protocols for efficient transposon mutagenesis in *Msmeg*^23^, which also allowed us to perform the first genome-wide identification of *in vitro* essential genes in this species. Interestingly, we found that the vast majority (90%) of *Msmeg* essential genes had a growth-required mutual ortholog in *Mtb*. Moreover, our low iron screens identified candidate genes for recognition, transport or utilization of siderophores, as well as new siderophore-independent low iron fitness mechanisms. For validation, we constructed a directed knockout of one of our hits, *msmeg_3635* (a homologue of a ferric iron ABC permease), and found that this gene is indeed important during *Msmeg* iron starvation and appears to function independently of siderophores.

## Results

### Msmeg *in vitro* essential genes

**Figure 1:**
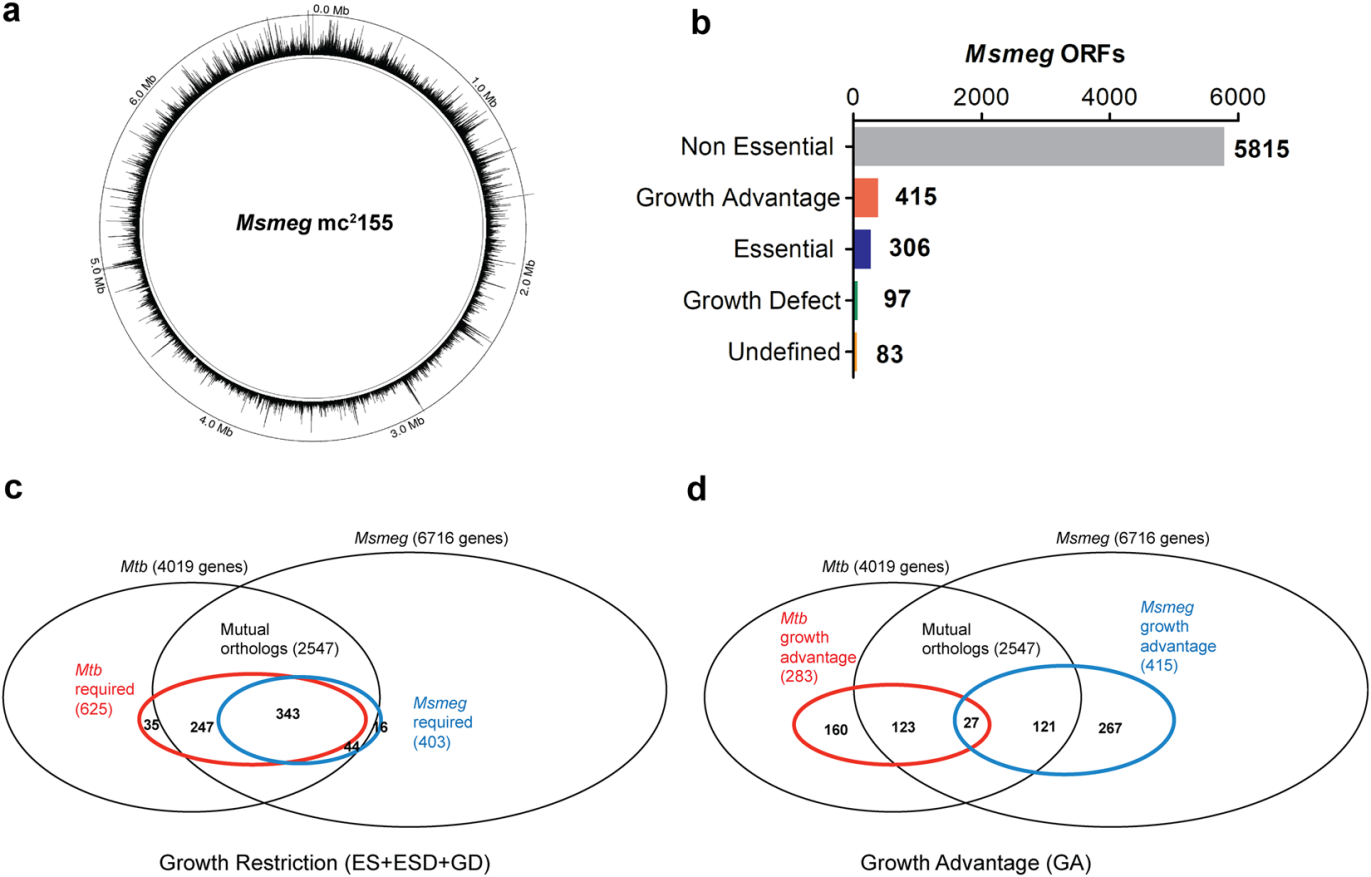
*Msmeg in vitro* growth requirement analysis. (**a**) Transposon insertion counts across the *Msmeg* genome (Middlebrook 7H10-selected library). The height of the black bars represents the number of insertion counts at the respective genome site. (**b**) Definition of the *Msmeg* wt *in vitro* requirement for growth (Middlebrook 7H10-selected library). 5815 genes were identified as non-essential (NE) for growth, 415 as causing growth advantage when disrupted (GA), 306 as essential (ES), and 97 as causing growth defect when disrupted (GD). 83 genes were not defined. (**c**) Venn diagram illustrating *Msmeg* required genes (ES and GD) and *Mtb* required genes (ES, GD and ESD, the latter genes with essential domains) as defined by DeJesus *et al*.^30^, relative to the entire pool of *Msmeg* (6716) and *Mtb* (4019) genes and their mutual orthologs (2547). *Msmeg* (403) and *Mtb* (625) required genes are shown within the blue and red circle, respectively. 343 genes are required in both species, 44 (*Msmeg*) and 247 (*Mtb*) required genes have a non-required mutual ortholog in the other species, and 16 (*Msmeg*) and 35 (*Mtb*) required genes do not have mutual orthologs in the other species (**d**) Venn diagram illustrating *Msmeg* and *Mtb* GA (causing growth advantage when disrupted) genes, as described for (**c**). *Msmeg* (415) and *Mtb* (283) GA genes are shown within the blue and red circle, respectively. 27 genes cause growth advantage in both species, 121 (*Msmeg*) and 123 (*Mtb*) GA genes have a non-GA mutual ortholog in the other species, and 267 (*Msmeg*) and 160 (*Mtb*) GA genes do not have mutual orthologs in the other species.

Transposon mutagenesis, via mycobacteriophage ϕMycomarT7, has been less efficient in *Msmeg* than other mycobacterial species^24^. By systematically altering our transduction protocols we were able to obtain high-density transposon libraries also in *Msmeg* mc^2^155^23^. The *Himar1* transposon of ϕMycomarT7 inserts relatively randomly in the 77,755 TA dinucleotides present in the *Msmeg* genome. To confirm good representation, we selected a library of ~500,000 *Msmeg* mutants selected on Middlebrook 7H10 and performed Tnseq of three independent DNA libraries. We found an average of 1 345,678 unique template counts (transposon insertion counts per TA site), covering 78% of the TA sites in the genome, with an average count of 22 per TA (excluding TA’s with zero insertions). Transposon insertions were evenly distributed throughout the genome (Fig. 1a).

Tnseq allows us to categorize genes by their requirement for growth^25^. Using a Hidden Markov Model incorporated into the TRANSIT platform^26^, we identified 306 *Msmeg* genes as essential (ES) for growth, 97 as causing growth defect (GD) and 415 as causing growth advantage (GA) when disrupted, and 5815 as non-essential (NE) (Fig. 1b, Dataset 1a). 83 genes were undefinable (N/A), due to insufficient number of TA sites present to robustly call their requirement status. As expected, we found insertions throughout two regions that are know to be copies of each other, ranging from *msmeg_1002*–1059 and *msmeg*_2282–2339^27,28^. In addition, we discovered a novel region that is probably present in more than one copy, ranging from *msmeg_4926*–4946 (see Supplementary Fig. S1). Interestingly, this region contains genes encoding F_1_F_0_ ATP synthase and one of three native copies of rRNA operons (*rrn*) (the *rrn* copy number is relevant for maximum growth rate^29^). As expected, the duplicated regions were over-represented in GA genes (67% of total genes within the duplicated regions, 9 times more than would be expected by random). Because there are additional copies of each gene in these loci, we cannot draw any conclusions about their actual requirement.

### *Msmeg* and *Mtbin vitro* essential genes compared

Differences and similarities in *Msmeg* and *Mtb* essential processes may illuminate our interpretation of data across species and influence our choice of study organism. *Msmeg* mc^2^155 shares 2547 mutual orthologous genes (meaning each gene in one species is the best match for the ortholog in the other species, with BLAST E-value < 10^−10^, Dataset 1b) with *Mtb* H37R*v*. We found that nearly all (96%) of the *Msmeg* required (ES and GD) genes had a mutual ortholog in *Mtb*. 90% of these genes were required also for optimal *Mtb in vitro* growth (as defined by DeJesus *et al*.^30^, note that Dataset 1a also lists the genetic requirement of *Mtb* mutual orthologs as defined by Griffin *et al*. and Zhang *et al*.^31,32^) (Fig. 1c), indicating that, under the given conditions, *Msmeg* and *Mtb* largely depend on the same mechanisms for growth. On the contrary, only 6% of *Msmeg* GA genes had a GA ortholog in *Mtb*, and the majority (64%) of the *Msmeg* genes providing growth advantage when disrupted were species-specific (i.e. no mutual ortholog) (Fig. 1d).

Orthologous genes that *differ* in requirement between *Msmeg* and *Mtb* may represent differences in niche or suggest alternative pathways (Dataset 1a). For instance, the thiamin biosynthesis pathway is essential in *Mtb*, but dispensable in *Msmeg*. Furthermore, genes within the *esx-3* gene cluster, required for mycobactin-mediated iron uptake, are essential in *Mtb* when cultured in standard (Middlebrook 7H10) medium but dispensable in *Msmeg* grown under similar conditions^17,33^. In *Msmeg*, a second iron acquisition system, exochelin, does not require ESX-3^17^. Thus, functional redundancy between exochelin and mycobactin might allow ESX-3 components to be disrupted in *Msmeg* but not *Mtb*

### Knockout mutants defective in siderophore-mediated iron acquisition

**Figure 2:**
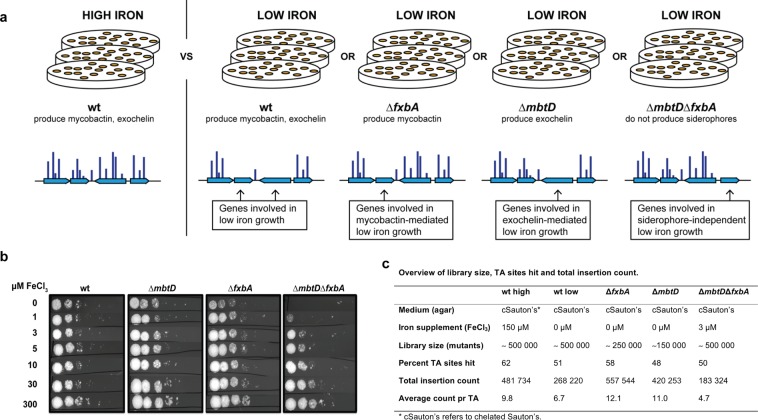
Schematic overview of screen for low iron fitness genes. (**a**) A transposon mutant library of *Msmeg* wt selected on high iron levels was compared to low iron-selected transposon libraries of *Msmeg* wt, Δ*fxbA* (exochelin knockout), Δ*mbtD* (mycobactin knockout) and Δ*fxbA*Δ*mbtD* (double siderophore knockout) to identify and categorize genes involved in low iron growth. The amplitude of the blue vertical bars represents the hypothetical number of transposon insertions counted at the given TA dinucleotide site. (**b**) *Msmeg* wt, Δ*fxbA*, Δ*mbtD* and Δ*fxbA*Δ*mbtD* mutants were prewashed in chelated Sauton’s before diluted and spotted on chelated Sauton-based agar plates with increasing concentrations of FeCl_3_. (**c**) Overview of library size, selective conditions, TA sites hit and total insertion count of the five sequenced libraries of the screen for low iron genes.

We have shown that *Msmeg* is suitable for genome wide phenotypic screening and uniquely apt to study mycobacterial low iron fitness mechanisms. We sought to identify and categorize *Msmeg* genes required for exochelin and/or mycobactin independent low iron survival by screening in strains deficient for siderophores (Fig. 2a). When one pathway is disrupted by a gene knockout, genes in that pathway are no longer likely to be essential, since the pathway itself no longer functions. However, genes in a compensatory or parallel pathway will become essential to rescue cells. This means that genes important for the mycobactin pathway are likely to be essential in low iron for cells unable to produce or utilize exochelin as the redundant siderophore is missing, and vice versa. Deletion of *fxbA* (*msmeg_0014*, a formyl transferase involved in synthesis of exochelin^34^) and *mbtD* (*msmeg_4512*, a polyketide synthase required for the synthesis of mycobactin^35^) previously abolished exochelin and mycobactin pathways, respectively^17^. Thus, we constructed *Msmeg* strains deficient in exochelin (*∆fxbA*), mycobactin (*∆mbtD*) and both siderophores (Δ*mbtD*Δ*fxbA*). The genetic deletions had the predicted effect; the exochelin (∆*fxbA*) and mycobactin (∆*mbtD*) deficient single mutant grew on low iron, however, the Δ*mbtD*Δ*fxbA* siderophore null mutant did not (Fig. 2b). This confirms that siderophores are fundamental for growth under the given condition. All strains grew in iron replete medium (Fig. 2b).

We found that the minimal amount of iron needed to rescue the siderophore null strain (Δ*mbtD*Δ*fxbA*) was between 1 and 3 μM, where iron acquisition must occur independently of siderophores through porins, yet-to-be-discovered transporters, or alternative iron acquisition mechanisms (Fig. 2b). Therefore, genes that become essential in siderophore null cells grown with 3 μM iron may function in a novel iron uptake pathway, independently of siderophores, or be required for survival by other means. For the optimal outcome of our low iron screens we therefore selected wild type (wt), exochelin null (*∆fxbA*), and mycobactin null (*∆mbtD*) libraries in the absence of iron supplementation, and the siderophore null (∆*mbtD*∆*fxbA*) library in the presence of 3 μM FeCl_3_ supplement (Fig. 2c).

### Differential growth analysis of high and low iron selected transposon libraries

**Figure 3:**
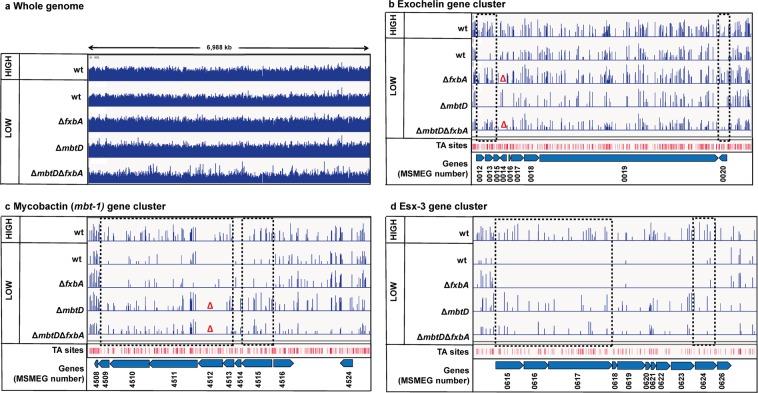
Validation of screen for low iron fitness genes. Distribution of transposon insertion counts (blue vertical bars) for all sequenced libraries. (**a**) Whole genome. Insertion counts in log scale of 1–1500. (**b**) Exochelin gene cluster. Insertion counts in log scale of 0–50. The red triangles indicate the knocked out gene (*msmeg_0014*, *fxbA*). (**c**) Mycobactin gene cluster. Insertion counts in log scale of 0–50. The red triangles indicate the knocked out gene (*msmeg*_4512, *mbtD*). (**d**) ESX-3 gene cluster. Insertion counts in log scale of 0–25. Plots created using IGV - distributed by the Broad Institute (http://www.broadinstitute.org/igv/). Black dashed boxes show genes significantly under-represented in insertion counts in one or more of the low iron libraries compared to the high iron library.

We generated transposon libraries of *Msmeg* wt, mycobactin null (Δ*mbtD*), exochelin null (Δ*fxbA*) and siderophore null (Δ*mbtD*Δ*fxbA*) mutants and selected on high or low iron media (Fig. 2c). Sequencing demonstrated that all libraries were adequately saturated with transposons (Fig. 3a, Dataset 1c). We expected that exochelin-related genes would become essential in the mycobactin null (∆*mbtD*) background in low iron. Indeed, genes in the exochelin pathway had few to no insertions in the ∆*mbtD* strain, while insertions in the same genes were readily apparent in ∆*fxbA* and ∆*mbtD*∆*fxbA* cells (Fig. 3b). Correspondingly, genes encoding enzymes required for mycobactin synthesis had lower counts in the exochelin null (∆*fxbA*) background than in ∆*mbtD* and ∆*mbtD*∆*fxbA* cells (Fig. 3c). Additionally, our previous finding that ESX-3 acts within the mycobactin utilization pathway was confirmed by the low insertion counts within the *esx-3* genetic cluster in the Δ*fxbA* but not Δ*mbtD* and ∆*mbtD*∆*fxbA* low iron selected libraries (Fig. 3d)^17^.

**Figure 4:**
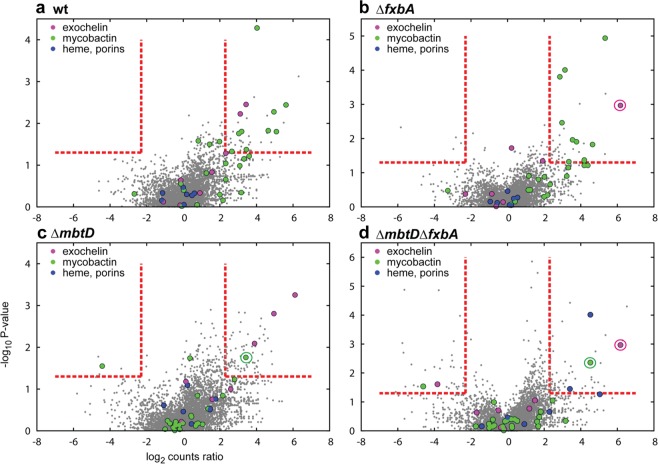
Identification and categorization of low iron fitness genes. Transposon insertion counts presented relative to counts ratio (control/experimental) per gene between *Msmeg* wt high iron control library and (**a**) *Msmeg* wt, (**b**) Δ*fxbA*, (**c**) Δ*mbtD* or (**d**) Δ*fxbA*Δ*mbtD* mutant low iron libraries (x-axis) and the corresponding P-values calculated by Mann Whitney U-test (y-axis). Genes previously known to be involved in mycobactin-mediated (green), exochelin-mediated (pink), or siderophore-independent (blue) iron uptake are color-coded. Red lines represent a cutoff of genes more than 5 fold under- (to the right) or over-represented (to the left) and with a P-value of less than 0.05. Genes knocked out are circled with pink (*fxbA*) or green (*mbtD*). In figure c is *msmeg_0019* out of scale with P-value 1.5 × 10^−9^ and relative counts ratio 1.478.

To identify new genes required for low iron growth, we normalized the insertion counts between the libraries and compared the ratio of counts on a gene by gene basis from the wt high iron control library to the low iron selected experimental libraries (Fig. 4). Here, ‘under-represented’ and ‘over-represented’ refer to genes with significantly lower or higher insertion counts compared to wt high iron, respectively. We found that a large fraction of previously known iron uptake genes (Dataset 1d), are among those significantly under-represented in our screens, confirming the synthetic lethality between mycobactin and exochelin pathways (Fig. 4a–d). As expected, genes involved in siderophore mediated iron uptake were dispensable in the double siderophore null background (Fig. 4d).

### Candidate genes required for low iron fitness

Comparing high and low iron-selected libraries allowed us to identify genes involved in iron acquisition as well as other processes directly or indirectly involved in low iron growth. For the wt library we found significant under-representation of 40 genes, of which 27.5% are previously known to be involved in siderophore-mediated iron uptake (Fig. 4a and Dataset 1e). We found several loci that had putative oxidation and reduction functions, all encoding proteins predicted to bind to metal co-factors. These enzymes might be required for basic cellular functions and bind iron even at very low iron concentrations. Other hits in our low iron screens included *moxR* (thought to chaperone insertion of metal cofactors into substrate molecules^36^), *msmeg_3121* (encodes a protein with homology to a regulator of iron-sulfur cluster assembly, SufR^37^), and *cysD* (sulfate adenylyltransferase, involved in sulfur metabolism, important for iron-sulfur cluster formation^38^), all relevant to iron as a co-factor. We also found predicted iron-responsive candidate genes, with putative binding sites for the iron dependent regulator IdeR^12,39–42^, such as *msmeg_6575* (*lipE*/*rv3775*), a putative β-lactamase/lipase.

### Candidate genes required for mycobactin-mediated iron acquisition

We know of few proteins involved in the recognition and transport of mycobactins across the mycobacterial outer membrane. Nor is the precise mechanism of ESX-3 in utilization of mycobactin-bound iron fully understood. Using the exochelin null library, we specifically screened for genes required for mycobactin-mediated iron uptake. We identified 36 significantly under-represented (Dataset 1f) and 6 over-represented genes (Dataset 1g). Nearly one-third of the under-represented genes were previously found to be involved in iron uptake via the mycobactin pathway (Fig. 4b). 16 hits not previously related to iron acquisition have *Mtb* orthologs and are, therefore, candidates genes for mycobactin-mediated iron uptake. In addition, genes involved in ATP synthesis (*atpA*, *atpB*) were under-represented in the exochelin null screen, consistent with an energy requirement for iron uptake (e.g. for the ATP-dependent transport of mycobactin via ABC transporter IrtAB).

### Candidate genes required for exochelin-mediated iron acquisition

We found that 72 genes were uniquely required in the strain lacking *mbtD* (Dataset 1h), while 7 were over-represented (Dataset 1i, Fig. 4c). Surprisingly, genes encoding proteins involved in import (FxuA-D), but not synthesis and export of exochelin (FxbA-C and ExiT), seem to be essential for low iron growth in ∆*mbtD* cells. It is possible that the production and export of exochelin from neighboring colonies may rescue transposon mutants unable to produce or export the siderophore. A similar trans-complementation of transposon mutants was previously seen in genes encoding synthesis of the *Yersinia pestis* siderophore yersiniabactin^43^. Interestingly, we do not see the same division of essentiality in genes of the mycobactin pathway. It might be that mycobactin is not as accessible to adjacent colonies as exochelin under these conditions, particularly since mycobactin is produced in an insoluble and membrane anchored form in addition to the secreted soluble form.

Nevertheless, we found genes that may be specific for exochelin utilization. For example *msmeg_4318* (encoding a hypothetical membrane transport protein), *msmeg_6063* (encoding a predicted iron uptake membrane protein), and *msmeg_6419* (encoding a conserved hypothetical predicted iron regulated protein^12,39^), are all required for *Msmeg* low iron growth in the absence of mycobactin synthesis and represent candidate exochelin uptake genes.

### Candidate genes required for siderophore-independent low iron fitness

Using the strain lacking both siderophores (Δ*mbtD*Δ*fxbA*) we found 57 significantly under-represented genes (Dataset 1j), and 42 over-represented genes (Dataset 1k, Fig. 4d). Genes involved in exochelin and mycobactin-mediated iron acquisition were not under-represented in this screen, which was expected, as the siderophore null (*∆mbtD∆fxbA*) mutant grows in a siderophore independent way. However, one gene known to be involved in heme uptake (*mmpL11*)^44,45^, and one involved in porin-mediated iron uptake (*mspA*)^46^, were found to be required for low iron growth in the absence of siderophores. This suggests that, while siderophores may be the predominant mechanism for *Msmeg* to access iron, under the given growth conditions, additional mechanisms can also take up the metal from the environment, albeit with lower efficiency. Novel genes of interest that may function in siderophore-independent iron uptake include *msmeg_5420*, which neighbors the predicted iron responsive iron permease *msmeg_5418*^39^. *Msmeg_5420* shows homology to the iron uptake gene *ywbN* of *Bacillus* *subtilis*^47^. Genes within a region spanning from *msmeg_1701* to *msmeg_1712* are under-represented in the ∆*fxbA* (*msmeg_1701*/*deoD*, *msmeg_1703*/*amiA1*), Δ*mbtD* (*msmeg_1705*) and Δ*fxbA*Δ*mbtD*-screens (*msmeg_1712*). *msmeg_1704-1712* encode a putative ABC transporter system, and this gene cluster might be important for low iron proliferation in a siderophore independent manner. Finally, two genes with homology to iron transport proteins, *msmeg_3635* and *msmeg_3636*, were significantly under-represented in the siderophore null screen (as were they in the Δ*mbtD*-screen, Dataset 1h and 1j), and we further characterized the role of *msmeg_3635* in mycobacterial low iron fitness below.

### *msmeg_3635* is required for *Msmeg* low iron fitness in the absence of siderophores

**Figure 5:**
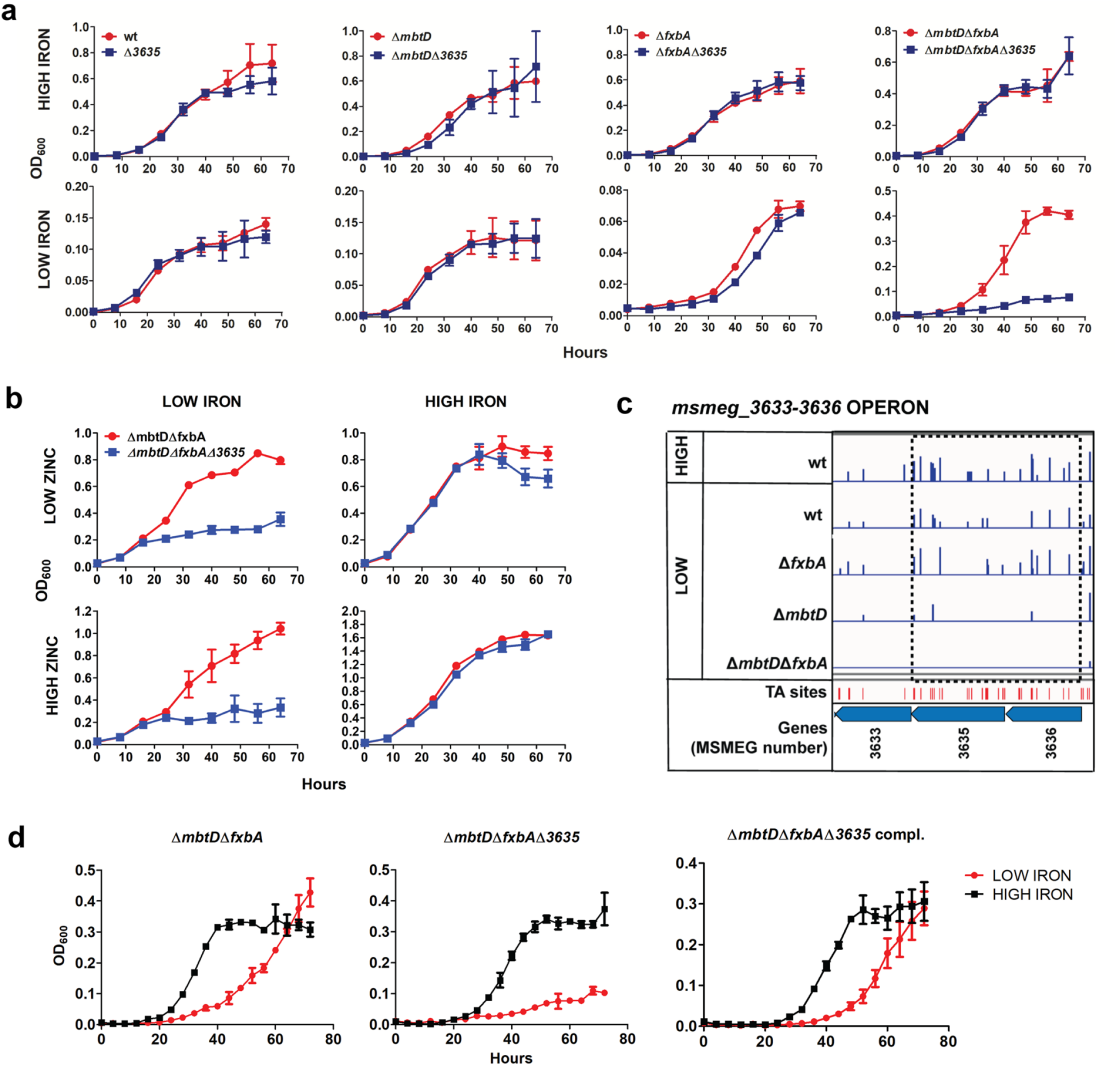
*msmeg*_*3635* is important for *Msmeg* siderophore-independent low iron growth. (**a**) Growth of *Msmeg* strains in high (upper panel) or low iron (lower panel) monitored over time (x-axis) by OD_600_ (y-axis). Error bars represent standard error from the mean of three biological replicas. To the Δ*fxbA*Δ*mbtD* and Δ*fxbA*Δ*mbtD*Δ*3635* curves, 3 μM FeCl_3_ was added in the low iron condition. To all other curves, 100 μM 2, 2′-bipyridine was add to low iron, and 150 μM FeCl_3_ was added to high iron condition. (**b**) Growth of Δ*fxbA*Δ*mbtD* and Δ*fxbA*Δ*mbtD*Δ3635 in low iron, low zinc (upper left), low iron, high zinc (lower left), high iron, low zinc (upper right), and high iron, high zinc (lower right). For all curves; low iron was supplemented with 3 μM FeCl_3_, low zinc with 0 μM ZnSO_4_, high iron with 150 μM FeCl_3_, and high zinc with 3.67 μM ZnSO_4_. (**c**) Distribution of transposon insertion counts (blue vertical bars) for all sequenced libraries in the *msmeg_3630-3636* operon. Insertion counts in log scale of 0–75. Plot created using IGV - distributed by the Broad Institute (http://www.broadinstitute.org/igv/). The black dashed box shows genes significantly under-represented in insertion counts in one or more of the low iron libraries compared to the high iron library. (**d**) Complementation of the Δ*fxbA*Δ*mbtD*Δ*3635* low iron phenotype. *Msmeg* Δ*mbtD*Δ*fxbA*, Δ*mbtD*Δ*fxbA*Δ*3635* and Δ*mbtD*Δ*fxbA*Δ*3635* compl. were grown in either high (150 μM FeCl_3_) or low (1.5 μM FeCl_3_) iron.

*msmeg_3635* belongs to a three-gene operon consisting of *msmeg_3633* (ATP binding protein), *msmeg_3635* (permease) and *msmeg_3636* (periplasmic protein), encoding the components of a putative ferric iron ABC transporter (Fig. 5c, Dataset 1a). To test whether this predicted transport system is indeed required for growth under low iron conditions, we disrupted it by constructing *msmeg_3635* deletions in wt and siderophore mutant backgrounds. The Δ*msmeg_3635* mutants showed impaired growth in low iron in the absence of both siderophores, but not in wt or single siderophore null backgrounds (Fig. 5a). All mutants grew at a rate similar to wt in high iron medium. Zinc, another ion required for optimal bacterial growth, is not able to rescue the double siderophore null mutant, suggesting the putative ABC transporter is iron-specific (Fig. 5b). Low iron growth of the Δ*mbtD*Δ*fxbA*Δ*3635* strain could be rescued by expression of the intact transport operon (Fig. 5d). Together, these results suggest that this putative transport system may translocate, or aid in translocation of, iron across the cell membrane in a manner independently of siderophore pathways.

## Discussion

Iron is an essential nutrient for bacteria, and is especially critical during host infection where iron itself is limited. *Mtb* depends on iron acquisition genes for virulence in animal models^2–5^, however, it is worth noting that Tufariello *et al*. found that the essentiality of *Mtb* mycobactin during mouse infection depended on host genotype^19^. Even so, many aspects of mycobacterial growth under iron limited conditions remain unknown, and the presence of a second high affinity iron uptake pathway in *Msmeg* allowed us to define a network of factors critical for mycobacterial low iron fitness. We identified novel genes potentially required for recognition, uptake or utilization of mycobactin and exochelin. Over all, nearly half (46%) of the low iron genes we identified had an ortholog in *Mtb*. However, when we look at the genes we found specifically required for the exochelin and mycobactin pathways, 32% and 72%, respectively, had *Mtb* orthologs. This reflects that the mycobactin pathway, but not the exochelin pathway, is present in *Mtb*. We also identified new genetic clusters and operons possibly involved in low iron growth independently of siderophores. To validate our screen, we investigated one such operon, *msmeg_3633*-*36*, encoding a putative ferric iron ABC transporter (with orthologs in *M*. *gilvum*, *M*. *vanbaalenii*, *M*. *thermoresistible*, and a multitude of other mycobacterial species, but not in *Mtb*). By disrupting the transporter we confirmed its involvement in siderophore independent low iron fitness. MSMEG_3635, encoded by the gene we knocked out, shares homology with the ferric iron transport system permease protein SfuB. In *Serratia marcescens*, SfuB was shown to be part of a putative transporter that enabled a siderophore-deficient strain of *Escherichia* *coli* K-12 to grow in iron-limited medium^48^, suggesting a conserved role for this protein in bacterial iron uptake.

Along with genes required for low iron growth, we found a surprising number of genes that became dispensable in low iron, as evidenced by over-representation in transposon libraries. This was particularly true when both siderophore pathways were deleted (Fig. 4), and might therefore be an effect of higher selective pressure in extremely iron-restricted environments. Some of the over-represented genes encode transcriptional repressors, and their absence might increase the expression of beneficial proteins. Interestingly, *mbtT* and *fxuB* (involved in mycobactin synthesis and exochelin import, respectively^34,35^) were over-represented in the Δ*mbtD* and/or Δ*mbtD*Δ*fxbA* screen(s). Both these genes had very few insertion counts in the wt high iron control library but not the respective low iron libraries. Their disruption might be toxic to the cells when iron is plentiful, perhaps by creating harmful intermediates or causing toxic siderophore accumulation, as previously seen for mycobactin^49^.

*Msmeg* has previously been central for our understanding of conserved mycobacterial functions (exemplified in^50–52^), though, as an environmental organism, it likely must adapt to a broader range of environmental stresses than the pathogen *Mtb*. It was therefore surprising to find that, under the given conditions, *Msmeg* and *Mtb* (as defined by DeJesus *et al*.^30^) largely depended on the same genes for optimal growth. In fact, only 4% of the *Msmeg* required genes did not have an *Mtb* mutual ortholog, a very small fraction considering the *Msmeg* genome contains 4169 (62%) genes with no *Mtb* mutual ortholog (albeit, some of the ‘non-mutual-orthologs’ will have partial or ambiguous *Mtb* orthologs). This large overlap (96%) in essential genes between the two species might reflect the reliance on a smaller set of conserved house-keeping genes required in a nutrient-rich, “non-stress” environment, like our test condition. Perhaps, if we subjected *Msmeg* to other growth conditions, more closely resembling its natural niches, we would find a larger fraction of species-specific genes required for growth. On the contrary, the majority (64%) of *Msmeg* GA genes (prompting growth advantage when disrupted) did not have a mutual orthologs in *Mtb*, in fact, only 6% of *Msmeg* GA genes had a GA *Mtb* mutual ortholog. Among the 6% mutual GA genes were several putative transcriptional regulators; their disruption might increase expression of genes benefiting growth under the given conditions. Many of the *Msmeg* GA genes were found within duplicated genome regions, partially explaining the difference in growth advantage seen between the two species. Also, the *Mtb* screen by DeJesus *et al*. was based on libraries selected in the presence of oleic acid supplement^30^, while our *Msmeg* library was not, potentially causing differences in genetic requirement between the two species.

Among genes that we found non-essential in *Msmeg*, but required in *Mtb*, were genes encoding thiamine and ATP biosynthesis (we have already mentioned the ESX-3 secretion system). Interestingly, *Mtb* does not appear to have a thiamine salvage and transport/uptake system^53^, while *Msmeg* encodes a putative thiaminase II (*msmeg_3478*), potentially involved in salvage of thiamine intermediates and thus rescue of *de novo* thiamine synthesis mutants^54^. A region we discovered to be present in more than one copy (*msmeg_4926*-4946) encodes F_1_F_0_ ATP synthase, probably explaining the non-essential nature of ATP synthesis in *Msmeg*. Actually, F_1_F_0_ ATP synthase was previously found essential in *Msmeg*, however, that was after knocking out both copies of *atpD*^55^. Taken together, the remarkable overlap in *Msmeg* and *Mtb in vitro* required genes, as well as the possibility to dissect differentially essential pathways using the non-pathogenic fast-growing strain, in our opinion, strengthen *Msmeg* as a model organism to study basic mycobacteriology and anti-mycobacterial drug discovery.

In summary; iron plays a central role in bacterial metabolism. Because of its scarcity, bacteria require highly avid molecules to adapt to iron limitation. Paradoxically, too much iron is toxic. Thus, these organisms have developed complex mechanisms to take up iron, regulate its uptake and maintain growth in iron-scarce host environments. Our work suggests that, in mycobacteria, several proteins have evolved to play critical roles in sustaining fitness when iron is low, and may propose attractive targets for new drugs.

## Methods

### Strains and growth conditions

*M*. *smegmatis* mc^2^155^56,57^ was cultured in Middlebrook 7H9 (BD Difco) medium supplemented with 0.2% glycerol, 0.05% Tween 80, and 10% albumin-dextrose-catalase (ADC) (5% [wt/vol] bovine serum albumin fraction V, 2% [wt/vol] dextrose, 145.5 mM NaCl, 0.003% [wt/vol] catalase), unless otherwise stated. *E. coli* DH5α was used for cloning and grown in LB medium. Kanamycin was added to 20 μg/ml or 50 μg/ml, and zeocin to 50 μg/ml or 25 μg/ml, in mycobacteria and *E*. *coli*, respectively. Hygromycin and gentamicin were added to 50 μg/ml and 7.5 μg/ml in mycobacteria, respectively. Liquid medium for *Msmeg* growth curves was prepared by adding 100 μM 2, 2′-bipyridine (Alfa Aesar) or 150 μM FeCl_3_ to chelated Sauton’s (prepared as previously described, using Chelex 100 resin from Bio-Rad^17^) for low or high iron, respectively. Intermediate iron medium (chelated Sauton’s with 1.5 or 3 μM FeCl_3_) did not contain 2, 2′-bipyridine. *Msmeg* transposon mutant library for *in vitro* gene requirement analysis (essential genes) was selected on Middlebrook 7H10 (BD Difco) agar medium supplemented with 0.5% glycerol, 0.1% Tween 80, and 10% ADC. *Msmeg* transposon mutant libraries for low iron gene requirement analysis were selected on agar medium prepare with chelated Sauton’s mixed with bacto agar (BD Difco) (see Supplementary SI Methods). All low iron media were prepared using plasticware.

### Construction of mutant strains

*Msmeg* mutant strains were constructed by recombineering, replacing the gene in question with an antibiotic resistance marker, using strains expressing the mycobacteriophage recombinases gp60 and gp61 on a nitril-inducible, counter-selectable plasmid or on the acetamide-inducible plasmid pJV53^58^. Details for construct preparations and selection processes are contained in Supplementary SI Methods^59,60^.

### Generation of *Msmeg* transposon mutant library

*Msmeg* transposon mutant libraries were prepared using the ϕMycomarT7 phagemid as recently described^23^.

### Preparation of libraries for Illumina sequencing

Total DNA of the transposon mutant libraries was purified using Masterpure DNA Purification kit (Epicentre). DNA fragmentation, end repair, A-tailing and adapter ligation was performed as previously described^32^. Transposon junctions were amplified by PCR and 200–400 bp fragments were isolated from gel and sequenced by Illumina GAII instrument.

### Genetic growth requirement analysis

The gene requirement calls were made using a Hidden Markov Model^25^, as implemented in TRANSIT^26^. The raw read counts mapping to each TA site in the *Msmeg* mc^2^155 genome (*Msmeg* mc^2^155_tamu) were reduced to unique templates counts by grouping based on nucleotide barcodes embedded in read 2^61^. Template counts at each TA site were exponentially scaled (f(t) = t^1.5^) so that the histogram of template counts better matches a Geometric distribution based on a Q-Q (quantile-quantile) plot, which is an assumption built-in to the HMM. The HMM uses 4-states, with labels ES (essential), GD (growth-defect), NE (non-essential), and GA (growth-advantage), and the likelihood that a TA site will be labeled with each state is based on differences between local read-count and the global average. The HMM was run on the Middlebrook 7H10-selected wt transposon insertion sequencing dataset, and the state labels of each site were post-processed to determine the majority label for each gene (ES, GD, NE, or GA), excluding TA sites in the N-terminal 10% and C-terminal 10% of the ORF.

### Differential growth analysis

The control library (wt selected on high iron) was compared to wt, Δ*mbtD* and Δ*fxbA* and Δ*mbtD*Δ*fxbA* libraries selected on low iron levels by determining the ratio of transposon insertion counts for the middle 94% of each gene. P-values were calculated by Mann-Whitney U test, treating the insertion counts as non-parametric distributions. A null hypothesis was used assuming the distribution of transposon insertion counts were similar for the respective genes between the two compared conditions. Promising candidates involved in iron acquisition were searched for within a list of genes with a P-value less than 0.05 (two tailed test) and a counts ratio of more than 5.

### Growth curve experiments

For *Msmeg*, cultures were grown in Middlebrook 7H9 for 2–3 days before washed once in chelated Sauton’s and diluted to OD_600_ 0.01 or 0.05 in the appropriate medium. Growth was monitored as 200 μl cultures (in triplicates) in microplate honeycomb wells using a Bioscreen growth curve reader (Oy Growth Curves Ab Ltd.).

## Supplementary information

Supplementary Info

Supplementary Methods and Figure S1

## Data Availability

All data generated during this study are included in this published article (and its Supplementary Information files).
