# Supplementary material for: Genome-wide Phenotypic Profiling Identifies and Categorizes Genes Required for Mycobacterial Low Iron Fitness
Source: Sci Rep. 2019 Aug 6;9:11394. doi: 10.1038/s41598-019-47905-y (PMC6684656; doi:10.1038/s41598-019-47905-y)
Supplement: Supplementary file 1 — Supplementary Info [file 41598_2019_47905_MOESM1_ESM.pdf]

Genome-wide Phenotypic Profiling Identifies and Categorizes Genes Required for Mycobacterial Low Iron Fitness

Marte S. Dragset[1,2,3,*], Thomas R. Ioerger[4], Yanjia J. Zhang[2], Mali Mærk[1], Zekarias Ginbot[1], James C. Sacchettini[5], Trude H. Flo[1], Eric J. Rubin[2,+], Magnus Steigedal[1,2,6,+]

Supplementary Methods

Strains and growth conditions. Low and high iron agar plates for selecting mutant libraries were prepared by mixing ~60°C concentrated chelated Sauton's (dissolving Sauton's ingredients in 0.75 L dH_2O as opposed to 1 L) with ~60°C 250 ml concentrated agar solution (15 g agar noble (BD Difco) in 250 ml iron free water boiled in a microwave in an appropriate plastic container until dissolved). 1 g $MgSO_4 \cdot 7H_2O$, 0.1% Tween-80 (both prepared using iron-free water), and the desired concentrations of $FeCl_3$ and kanamycin were added before plates were poured. Iron free water was prepared by stirring 10 g Chelex 100 (Bio-Rad) in 1 l dH_2O for two days room temperature before filter sterilized.

Construction of *Msmeg* mutant strains. Primer sequences are available upon request. *Msmeg* mutant defective in mycobactin synthesis (Δ*mbtD*) was created by replacing *msmeg_4512*, encoding a polyketide synthase, with the zeocin resistance gene *Sh ble*. The 200 bp flanking the 3' and 5' ends of *mbtD*, separated by a linker containing the appropriate restriction sites was ordered in a pUC57-simple vector from Genscript (USA). *Sh ble* was cloned in between the flanks and the whole cassette (flanks with resistance gene) was amplified by PCR. Allelic exchange of *mbtD* was performed by transformation of the latter PCR product into *Msmeg* expressing the mycobacteriophage recombinases gp60 and gp61 on a nitril-inducible, counter-selectable plasmid. The mutant was confirmed by southern blot before the recombineering vector was removed by sucrose counter-selection. The Δ*mbtD*Δ*fxbA* mutant was created as described for Δ*fxbA* in [60], using the Δ*mbtD* strain as template

for recombineering. *msmeg_3635* knockout mutants were created as described above for Δ*mbtD*, but with gentamicin resistance as a selection marker. Allelic exchange of *msmeg_3635* was performed by transforming amplified gentamicin resistance gene with flanks into *Msmeg* expressing pJV53 according to [58], with the exception that 1-1.5 μg PCR-amplified substrate was used. The mutants were confirmed by PCR and pJV53 was removed by re-patching positive clones on to plates containing gentamicin. This resulted in mutants Δ*3635*, Δ*mbtD*Δ*3635*, Δ*fxbA*Δ*3635* and Δ*mbtD*Δ*fxbA*Δ*3635*. The antibiotic resistance cassette replacing the genes in question was not removed from the abovementioned mutants. To rescue the Δ*mbtD*Δ*fxbA*Δ*3635* low iron phenotype, the *msmeg_3636-3635-3633* operon was inserted downstream of the *Pm* promoter in pMDX [59]. Due to difficulties with cloning the full operon in one operation, this was done in a stepwise manner, resulting in pMM42. pMM42 was transformed into Δ*mbtD*Δ*fxbA*Δ*3635* mutant cells to create strain Δ*mbtD*Δ*fxbA*Δ*3635* compl.

Supplementary Figure

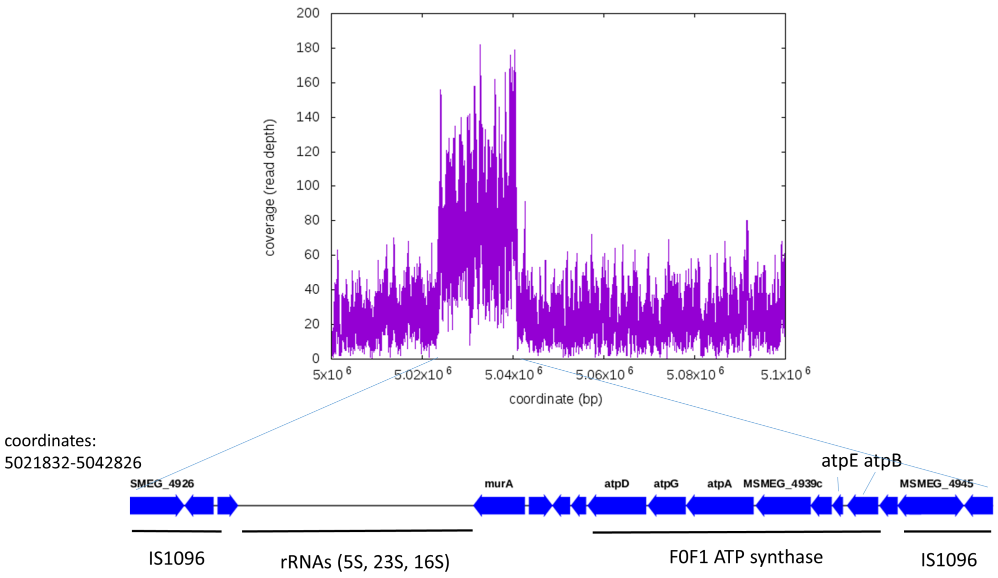

Supplementary Figure S1: Coverage (read depth) of coordinates 5021832-5042826. A shift in the read depth between *msmeg_4926–4946* suggests that there is more than one copy of this genome sequence present in *Msmeg* mc^2155_tamu.
